# The *MCPH7* Gene Product STIL Is Essential for Dendritic Spine Formation

**DOI:** 10.3390/cells14020062

**Published:** 2025-01-07

**Authors:** Tohru Matsuki, Hidenori Tabata, Masashi Ueda, Hideaki Ito, Koh-ichi Nagata, Yumi Tsuneura, Shima Eda, Kenji Kasai, Atsuo Nakayama

**Affiliations:** 1 Department of Cellular Pathology, Institute for Developmental Research, Aichi Developmental Disability Center, Kasugai 480-0392, Aichi, Japaneda@inst-hsc.jp (S.E.); 2 Department of Molecular Neurobiology, Institute for Developmental Research, Aichi Developmental Disability Center, Kasugai 480-0392, Aichi, Japan; tabata@inst-hsc.jp (H.T.); knagata@inst-hsc.jp (K.-i.N.); 3 Department of Pathology, Aichi Medical University School of Medicine, Nagakute 480-1195, Aichi, Japankkasai@aichi-med-u.ac.jp (K.K.); 4 Department of Neurochemistry, Nagoya University Graduate School of Medicine, Nagoya 466-8550, Aichi, Japan

**Keywords:** MCPH7, STIL, dendritic spine, ARHGEF7, Rac1, Cdc42

## Abstract

Dendritic spine formation/maintenance is highly dependent on actin cytoskeletal dynamics, which is regulated by small GTPases Rac1 and Cdc42 through their downstream p21-activated kinase/LIM-kinase-I/cofilin pathway. ARHGEF7, also known as ß-PIX, is a guanine nucleotide exchange factor for Rac1 and Cdc42, thereby activating Rac1/Cdc42 and the downstream pathway, leading to the upregulation of spine formation/maintenance. We found that STIL, one of the primary microcephaly gene products, is associated with ARHGEF7 in dendritic spines and that knockdown of *Stil* resulted in a significant reduction in dendritic spines in neurons both in vitro and in vivo. Rescue experiments indicated that the STIL requirement for spine formation/maintenance depended on its coiled coil domain that mediates the association with ARHGEF7. The overexpression of Rac1/Cdc42 compensated for the spine reduction caused by STIL knockdown. FRET experiments showed that Rac activation is impaired in STIL knockdown neurons. Chemical long-term potentiation, which triggers Rac activation, promoted STIL accumulation in the spine and its association with ARHGEF7. The dynamics of these proteins further supported their coordinated involvement in spine formation/maintenance. Based on these findings, we concluded that the centrosomal protein STIL is a novel regulatory factor essential for spine formation/maintenance by activating Rac and its downstream pathway, possibly through the association with ARHGEF7.

## 1. Introduction

Dendritic spines are small protrusions along neuronal dendrites originally identified by Santiago Ramón y Cajal more than a century ago [1,2]. It is generally believed that most spines host a single excitatory synapse, whereby spines are regarded as morphological surrogates for excitatory synapses that should otherwise be identified by electron microscopy [1]. Recent advances in in vivo imaging techniques have unveiled that spines dynamically change in number and shape during development and activity-dependent neural circuit remodeling [1]. Hence, spine dynamics is considered to be the morphological basis for the formation and remodeling of neural networks and is highly relevant to neural functions such as memory and learning. Naturally, the regulatory mechanisms underlining spine dynamics have been of great interest; yet, these mechanisms are not fully elucidated. Until now, however, numerous key molecules and major signaling pathways have been identified, including Rho members of the small GTPase superfamily. As molecular switches cycle between GTP-bound active forms and GDP-bound inactive forms, Rac1 and Cdc42 regulate the actin cytoskeleton through the p21-activated kinase (PAK)/LIM-kinase-I (LIMK-I)/actin depolymerizing factor cofilin pathway in local membrane dynamics [3], and this pathway is also considered responsible for spine morphology [4,5]. Among the various guanine nucleotide exchange factors (GEFs) that activate GTPases, ARHGEF7 (also known as ß-PIX), along with Kalirin-7 and Tiam1, is a Rac-GEF that has been well studied in relation to dendritic spine formation/maintenance [5,6]. The reduction in spines in cultured hippocampal neurons upon the introduction of dominant-negative ARHGEF7 or knockdown of *Arhgef7* [7,8] indicated that ARHGEF7 is required for Rac activation in the context of spine formation/maintenance. Recently, we reported that ARHGEF7 and PAK1 together associated with STIL, one of the centrosomal proteins, at the lamellipodia protrusion of motile cells and that STIL is involved in the ARHGEF7-mediated activation of cytoskeletal remodeling in cancer cells [9]. These findings prompted us to explore the possibility that STIL is also involved in ARHGEF7/Rac-mediated spine dynamics.

Here, we report that STIL is located in the dendritic spine, where it associates with ARHGEF7. The induction of chemical long-term potentiation (cLTP), which triggers spine formation and expansion, increased the amount of STIL in the dendritic spine and promoted their association with ARHGEF7. *Stil* knockdown resulted in a significant reduction in dendritic spines in both hippocampal neurons in vitro and cerebral cortical neurons in vivo. The introduction of Rac1 and Cdc42, known targets of ARHGEF7, into *Stil* knockdown neurons compensated for the reduction in spines. Impaired Rac activation was evidenced in cultured *Stil* knockdown neurons by fluorescence resonance energy transfer (FRET) experiments. These data revealed a novel and indispensable role for STIL in spine formation/maintenance and suggested that this role is mediated by association with ARHGEF7 and the activation of Rac GTPases.

## 2. Materials and Methods

### 2.1. Animals

All animals were handled in accordance with the protocol approved by the Animal Committee of the Institute for Developmental Research (approval # 2019-003R5). Timed-pregnant ICR wild-type mice were purchased from Japan SLC (Hamamatsu, Shizuoka, Japan) and were used for in vitro cell culture experiments and in utero electroporation.

### 2.2. Cell Culture

Both cortical and hippocampal neurons were prepared from embryonic day (E) 16.5 mouse embryos and maintained for appropriate periods in a neurobasal medium supplemented with 2% B27 and Glutamax-I (Thermo Fisher Scientific, Waltham, MA, USA). For quantitative RT-PCR analysis, the neurons (1–2 × 10^5^ cells per cm^2^) were infected with respective lentiviruses on 5 days in vitro (DIV) and harvested 3 days after the infection. For dendritic spine observation, the neurons (1–2 × 10^4^ cells per cm^2^) were cultured on poly-L-lysine-coated coverslips and infected with the respective lentiviruses on 5 DIV. The cultured neurons were maintained by replacing one-third of the culture media every 2 days until analyses.

### 2.3. Antibodies

We used the following antibodies for immunocytochemistry: anti-STIL (1:250, [10]), anti-PSD95 (1:1000, NeuroMab, Davis, CA, USA, K28/43), and anti-ARGEF7 (1:100, Santa Cruz, Dallas, TX, USA, sc-393184). Anti-β-Actin (1:5000, Merck, Darmstadt, Germany, A1978) was applied for immunoblotting, and anti-GFP (1: 600, Thermo Fisher Scientific, A10262) and anti-RFP (1:1000, Rockland Limerick, PA, USA, 600-401-379) were applied for immunohistochemistry. Alexa 647-conjugated phalloidin (1:1000, Abcam, Cambridge, UK, ab176759) was used to detect filamentous actin (F-actin). We also used following secondary antibodies: Alexa Fluor 488-conjugated goat anti-chicken IgG (H + L) antibody (Thermo Fisher Scientific, A11039), Alexa Fluor 647-conjugated donkey anti-chicken IgG (H + L) antibody (Jackson Immunoresearch, West Grove, PA, USA, 703-605-155), Alexa Fluor 488-conjugated goat anti-rabbit IgG (H&L) antibody (Thermo Fisher Scientific, A11034), Alexa Fluor 568-conjugated goat anti-mouse IgG (H&L) antibody (Thermo Fisher Scientific, A11031), and Alexa Fluor 568-conjugated goat anti-rabbit IgG (H&L) antibody (Thermo Fisher Scientific, A-11011),

### 2.4. Quantitative RT-PCR

Total RNA was extracted from the cultured neurons on 8 DIV using Isogen II (Nippon Gene, Tokyo, Japan). The cDNA template was prepared using ReverTra Ace qPCR RT Master Mix (TOYOBO, Osaka, Japan). Quantitative PCR analysis was performed using THUNDERBIRD SYBR qPCR Mix (TOYOBO) and a CFX96 Touch Real-Time PCR Detection System (Bio-Rad, Hercules, CA, USA) with the primers listed in Table 1. *Stil* expression levels were normalized to *Actb* levels and averaged across triplicate experiments using three independent samples.

### 2.5. Plasmid and Viral Vector Construction

Primers used for plasmid construction, along with shRNA target sequences, are listed in Table 1. HA-tagged wild-type and mutant human STIL were cloned individually into a pCAG vector. Lentivirus vectors for the expression of shRNAs and EGFP were developed by modifying pLL3.7 (#11795, Addgene, Watertown, MA, USA). First, the CMV promoter driving the EGFP expression of pLL3.7 was replaced with the human Synapsin I promoter to make the pLLS vector. The pLLS vector was further modified to express shRNA under the U6 promoter. The shRNA sequences for mouse *Stil* knockdown were based on the Sigma MISSION shRNA library provided by the RNAi Consortium (Boston, MA, USA). The pLLS vector was also utilized to express human STIL and STIL lacking the coiled coil domain (STIL ∆CC) instead of EGFP under the Synapsin I promoter. Lentivirus vectors expressing Rac1, constitutively active Rac1, Cdc42, or constitutively active Cdc42 were also developed by modifying the pLL3.7 vector. The CMV promoter and EGFP-coding region of pLL3.7 was replaced with the Chicken β-Actin promoter and a fragment encoding Rac1, Rac1 V12 (constitutively active Rac1 with G12V substitution), Cdc42, or Cdc42 V12 (constitutively active Cdc42 with G12V substitution) and, subsequently, T2A-RFP.

### 2.6. In Utero Electroporation (IUE)

For in utero electroporation, plasmid DNA was mixed with Fast Green dye in PBS and injected into one side of the lateral ventricles of E14.5 mouse embryos. Electrostimulation was applied to the embryos with 5 electric pulses (35 V 50 ms duration and 450 ms intervals) using a tweezers-type 5 mm platinum plate electrode (NEPAGENE, Ichikawa, Chiba, Japan, CUY650P5) and a NEPA21 electroporator (NEPAGENE). The mice were then raised until postnatal day (P) 30 for dendritic spine observation. Each mouse was sacrificed under anesthesia and perfused with 4% paraformaldehyde (PFA) in PBS. Isolated brains were embedded in 3% Agarose gel and were sectioned in the coronal plane at 80 µm-thickness with a VT1200S vibratome (Leica Microsystems, Wetzlar, Germany).

### 2.7. Immunocytochemistry and Immunohistochemistry

Immunocytochemistry was carried out according to our previous study [11]. Briefly, cultured hippocampal neurons were fixed in 4% PFA/modified Pagano solution (250 mM sucrose, 2.5 mM KCl, 2.5 mM magnesium acetate, and 25 mM HEPES (pH 7.4), [12] for 10 min at 25 °C. Fixed cells were treated with 0.1% saponin in PBS for 10 min at 25 °C for permeabilization and with 3% BSA/TTBS (TBS containing 0.1% Tween20) for 1 h at room temperature. Then, the samples were incubated with primary antibodies diluted with 3% BSA/TTBS overnight at 4 °C. On the next day, the samples were washed with TTBS three times and incubated with secondary antibodies diluted with 3% BSA/TTBS for 1 h at room temperature. To visualize F-actin, the samples were incubated with fluorescently labeled phalloidin diluted with 0.1% BSA/PBS for 30 min at 25 °C. For immunohistochemistry, 80 µm-thickness brain sections were incubated in blocking solution (TBS containing 5%BSA and 0.25% Triton X-100) for 1 h at 25 °C and incubated with anti-GFP antibodies overnight at 4 °C. The sections were washed with washing solution (TBS containing 0.25% Triton X-100) three times and incubated with secondary antibodies diluted with blocking solution for two hours at 25 °C. Both cell and tissue samples were mounted with VECTOR Shield anti-fade solution (Vector Laboratories, Inc., Burlingame, CA, USA).

### 2.8. Chemical Long-Term Potentiation (cLTP) Induction in Cultured Neurons

We induced cLTP in cultured hippocampal neurons following a previous study [13]. Neurons cultured on glass coverslips were treated with extracellular solution (125 mM NaCl, 2.5 mM KCl, 2.0 mM CaCl, 33.0 mM D-Glucose, and 25.0 mM HEPES (pH 7.4)) for 20 min at 25 °C. Then, the cells were incubated with cLTP induction solution (the extracellular solution supplemented with 200 μM Glycine, 20 μM Bicuculline, 3 μM Strychnine, and 0.5 μM TTX) for 10 min at 25 °C and were washed with the cLTP induction solution without Glycine for 30 min at 25 °C before fixation.

### 2.9. Spine Density Analysis

3D images of GFP-expressing cultured neurons and neurons in brain sections were captured by LSM880 confocal microscopy (Carl Zeiss, Oberkochen, Germany). The segments of dendrites for analysis were selected according to the following criteria: the starting point was at least 50 μm distal to the soma and distal to the first branching point, and the ending point was at least 20 μm distal to the starting point. Each z-stuck image file was reconstructed using Imaris software version 8.3 (Bitplane AG, Zurich, Switzerland) and was then used to calculate their spine densities.

### 2.10. Proximity Ligation Assay (PLA)

Cultured hippocampal neurons were fixed and processed according to the same methodology used for immunocytochemistry and were subjected to PLA. We performed PLA using a Duolink in situ PLA kit (MilliporeSigma, St. Louis, MO, USA) following the manufacturer’s protocol. After PLA, the samples were subjected to phalloidin staining to visualize F-actin. Both PLA and phalloidin signals were captured using LSM880 confocal microscopy.

### 2.11. Fluorescence Resonance Energy Transfer (FRET) Analysis

Cultured hippocampal neurons on 10 DIV were transfected with the lentivirus version of Raichu 1011x, which was kindly gifted by Dr. Matsuda at Kyoto University [9]. At 17 DIV, the neurons were fixed with 2% PFA/modified Pagano solution for 3–5 min at room temperature, which is the fixation condition applicable to FRET analysis [11], and were immediately subjected to the analysis. When necessary, cLTP was induced in the neurons just before the fixation. All FRET images were obtained using LSM 880 confocal microscopy as described previously [11]. The FRET ratio (YFP/CFP) images were processed using ZEN 3.4 software (Carl Zeiss, Jena, Germany). FRET efficiencies were calculated using the following formula: FRET efficiency (in %) = (1-prebleach CFP value/postbleach CFP value) × 100. The FRET ratio was normalized using the FRET efficiency.

### 2.12. Statistical Analysis

All the experiments were performed at least in triplicate. Comparisons between experimental groups including controls were analyzed using one-way ANOVA with Holm-Bonferroni post-hoc test. All statistical analyses were performed using OriginPro 2021 (OriginLab, Northampton, MA, USA). The error bars indicate the standard error of the mean (SEM).

## 3. Results

### 3.1. STIL Is in the Dendritic Spine and Is Closely Associated with ARHGEF7

**Figure 1:**
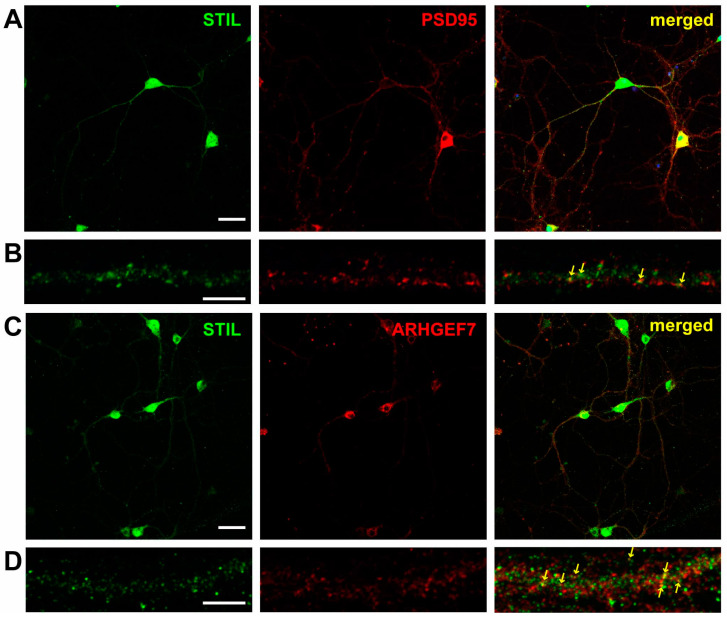
STIL co-localizes with PSD95 and ARHGEF7 along dendrites of cultured hippocampal neurons. (**A**) Representative images of hippocampal neurons stained for STIL (green) and PSD95 (red). (**B**) High magnification views of STIL and PSD95 distribution in a dendrite. Co-localization of STIL and PSD95 signals is indicated by arrows in the merged image. (**C**) Representative images of hippocampal neurons stained for STIL (green) and ARHGEF7 (red), along with a merged image. (**D**) High magnification views of STIL and ARHGEF7 distribution in a dendrite. Co-localization of STIL and ARHGEF7 signals is indicated by arrows in the merged image. Scale bars = 50 µm for (**A**,**C**) and scale bars = 5 µm for (**B**,**D**).

We first examined the subcellular distribution of STIL in primary cultured mouse neurons. As shown in Figure 1A, STIL was widely distributed in the cell body of neurons. At the same time, STIL signals were identified as puncta along the neurites, some of which co-localized with PSD95 signals (Figure 1B). This indicated that a portion of STIL was distributed in the dendritic spine. Next, we examined whether STIL located along neurites co-localized with ARHGEF7 and found that a portion of STIL co-localized with ARHGEF7 (Figure 1C,D). However, in co-immunoprecipitation (co-IP) experiments using crude neuronal lysates as well as synaptosomal fractions, little binding between endogenous STIL and ARHGEF7 was detected (Figure S1), while we have previously shown binding in cancer cells [14]. We hypothesized that the discrepancy between the results in neurons and cancer cells was due to the rare binding between STIL and ARHGEF7 in neurons since the abovementioned immunocytochemical observation suggests that only a portion of STIL associated with ARHGEF7 in cultured neurons.

**Figure 2:**
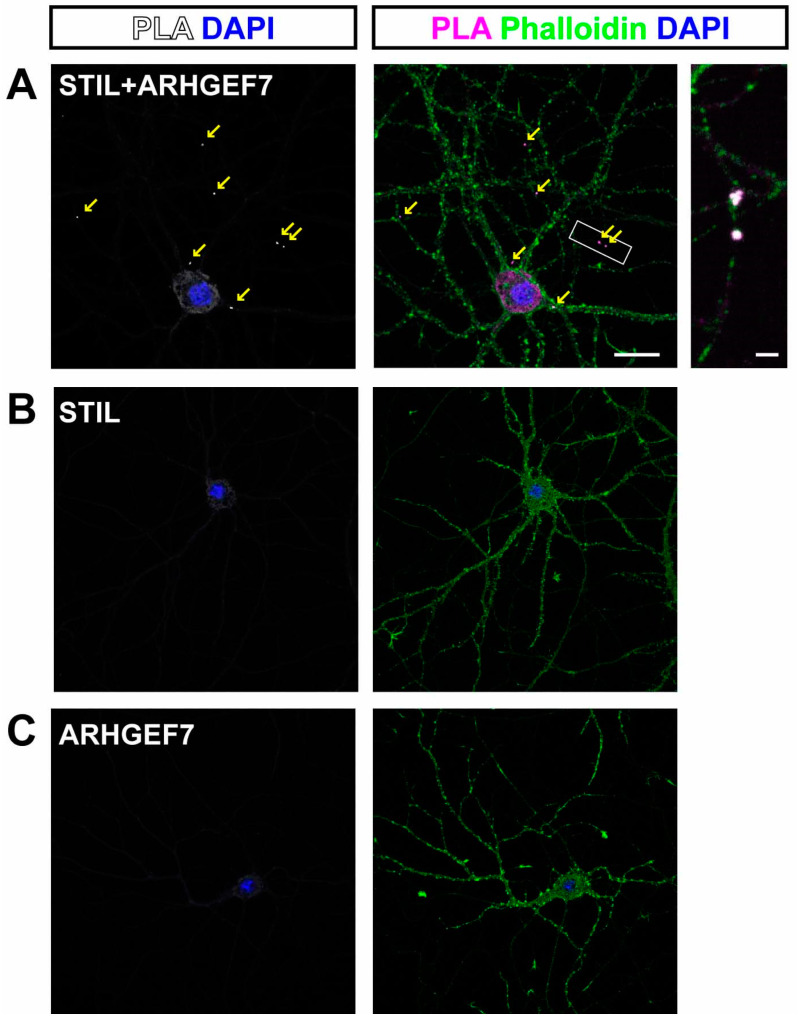
STIL associates with ARHGEF7 in dendritic spines of cultured hippocampal neurons. (**A**) Representative images of cultured hippocampal neurons subjected to proximity ligation assay (PLA). PLA signals originating from the proximity of STIL and ARHGEF7 are detected as red spots and are indicated by arrows (**left**). A green phalloidin-stained image is overlaid on the PLA image to show dendritic spines (**middle**). The PLA signals appear as white or magenta spots, indicating that they localize to the dendritic spine. The right image shows a magnified view of the boxed area in the middle. Scale bars = 20 µm (**middle image**) and 2 µm (**right image**). (**B**,**C**) Representative images of cultured hippocampal neurons in negative control experiments. No PLA signal is observed when antibodies against STIL (**B**) and ARHGEF7 (**C**) are applied independently.

To identify such rare binding, we applied the proximity ligation assay (PLA) [15], in which a PLA signal spot is formed when two proteins are within 40 nm of each other. PLA signal spots derived from the proximity of STIL and ARHGEF7 were observed, along the neurites, and most of the spots were co-localized with phalloidin signals, indicating that the spots were in the spine (Figure 2A). PLA signal spots were indeed rare, with up to a few identified in each neuron. In contrast, no signal spots were observed in control experiments, in which STIL or ARHGEF7 antibodies were applied alone (Figure 2B,C).

### 3.2. Stil Knockdown Results in the Reduction in Dendritic Spine Density In Vitro

**Figure 3:**
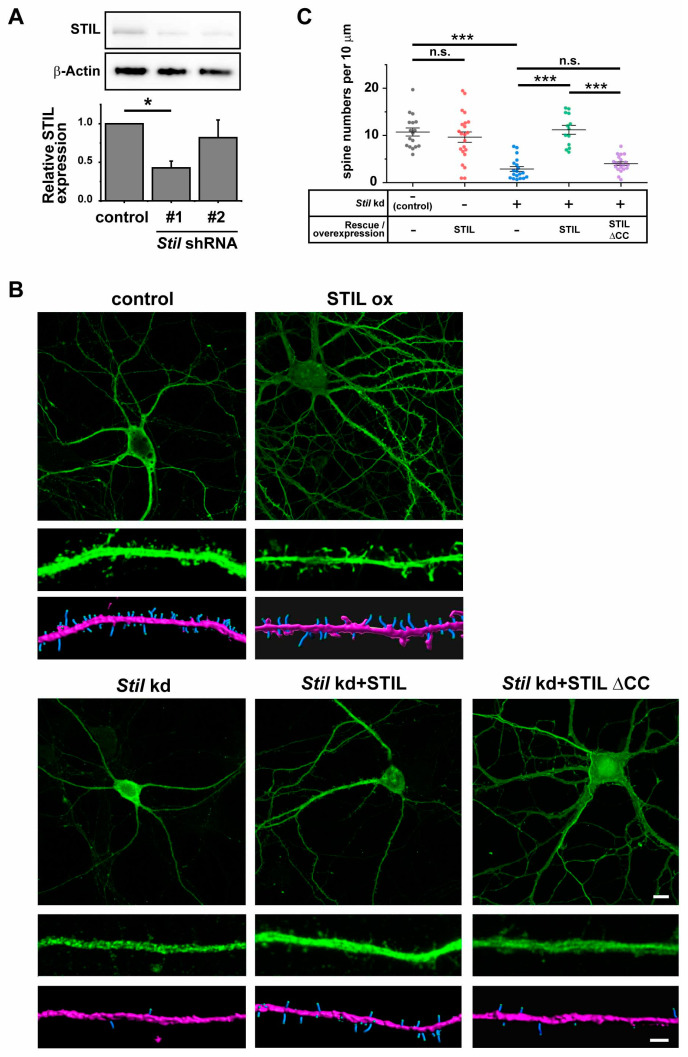
*Stil* knockdown causes dendritic spine reduction in cultured hippocampal neurons, which is rescued by knockdown-resistant human STIL but not by human STIL lacking the coiled coil domain. (**A**) Confirmation of *Stil* knockdown by *Stil* shRNAs. Proteins were extracted from cultured neurons infected with lentiviral vectors expressing *Stil* shRNA or control empty vectors, and STIL expression levels were examined by Western blotting in four independent experiments. A representative STIL band image is shown at the top, and an anti-β-Actin band image as loading control is shown at the middle. The signal intensity of the STIL band was corrected against the signal intensity of the β-Actin band, and a graph of the values compared to the control is shown at the bottom. * *p* < 0.01 (**B**) Representative images of a neuron overexpressing STIL and *Stil* knockdown neurons introduced with/without rescue vectors. Raw images of whole cells (**top panel**) and magnified dendrites (**middle panel**) and reconstructed images of dendrites (**bottom panel**) of hippocampal neurons cultured under various experimental conditions are shown. EGFP alone (control), STIL (STIL ox), *Stil* shRNA #1 (*Stil* kd), *Stil* shRNA #1 with knockdown-resistant STIL (*Stil* kd + STIL), or *Stil* shRNA #1 with knockdown-resistant STIL lacking a coiled coil domain (*Stil* kd + STIL ∆CC) was introduced into neurons. The spines and dendritic shafts are visualized in blue and pink, respectively, in the reconstructed images. Scale bars = 10 µm (**top panel**) and 2 µm (**bottom panel**). (**C**) Distribution of dendritic spine density (number of spines per 10 µm of dendrite) under each condition. Each dataset was obtained from dendrites in neurons analyzed in at least three experiments. Mean values: 10.721 (control, *n* = 17), 9.637 (STIL WT, *n* = 22), 8.158 (STIL ∆CC, *n* = 22), 2.877 (*Stil* kd, *n* = 19), 11.188 (*Stil* kd + STIL WT, *n* = 13), and 4.005 (*Stil* kd + STIL ∆CC, *n* = 20). *** *p* < 0.001. n.s., not significant.

To determine the functional relevance of STIL in the dendritic spine, we knocked down *Stil* in primary cultured neurons and examined its effect on dendritic spines. We designed two *Stil* shRNAs to knockdown mouse *Stil* and confirmed that only *Stil* shRNA#1 statistically significantly interfered with STIL protein expression in primary neurons (Figure 3A). The STIL protein band in Western blotting experiments appeared similarly weak, but calibration with β-Actin expression indicated that the effect of *Stil* shRNA #2 was not sufficient. Similar results were also obtained for *Stil* mRNA expression by qRT-PCR (the mean relative values of *Stil* expression; control = 0.88505, shRNA #1=0.53783, shRNA #2=0.68041, *p*-values; control vs shRNA #1 = *p* < 0.01, control vs shRNA #2 = n.s., *n* = 3). Therefore, we chose *Stil* shRNA#1 for further knockdown experiments. Prior to the knockdown experiments, we examined the effect of simple STIL overexpression on spines. As shown in Figure 3B,C, STIL overexpression (STIL ox) showed no significant effect on spine density, indicating that excess amounts of STIL neither promote nor suppress spine formation in cultured neurons. Then, we knocked down *Stil* by introducing the shRNAs into primary cultured hippocampal neurons at 5 DIV, and dendritic spines were analyzed between 14 and 17 DIV. *Stil* knockdown resulted in significant dendritic spine reduction in neurons (*Stil* kd); the mean dendritic spine density of *Stil* knockdown neurons was about 27% of that of control neurons (Figure 3C). The reduction was completely restored when neurons were co-infected with shRNA-resistant human *STIL* cDNA (Figure 3B,C, *Stil* kd + STIL). These results clearly indicate that STIL is essential for dendritic spine formation and/or maintenance in primary cultured neurons. We further wanted to evaluate whether the involvement of STIL in spine formation/maintenance might depend on its association with ARHGEF7. Therefore, we examined the rescue effect of shRNA-resistant STIL cDNA lacking the coiled coil (CC) domain that mediates association with ARHGEF7 [14]. As shown in Figure 3B,C, human STIL without the CC domain retained little rescue effect (*Stil* kd + STIL ∆CC). These results revealed that the CC domain of STIL was crucial for spine formation/maintenance and suggested that STIL requirement for spine formation/maintenance depends on its association with ARHGEF7. However, we cannot exclude the possibility that other functions mediated by the CC domain may be involved in the observed results.

### 3.3. Rac1 and Cdc42 Induction Can Compensate for the Reduction in Dendritic Spine Caused by Stil Knockdown

**Figure 4:**
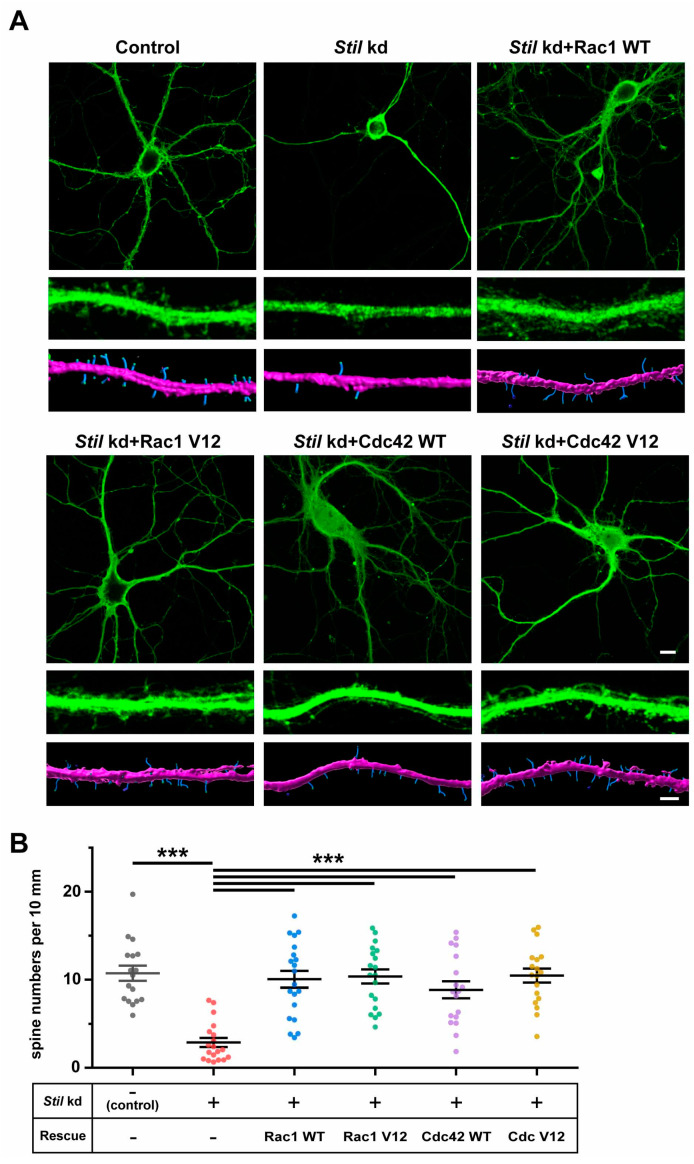
Rac1 and Cdc42 induction can compensate for the reduction in dendritic spines caused by *Stil* knockdown. (**A**) Representative images of control and *Stil* knockdown neurons with/without expressing wild-type or constitutively active Rac1/Cdc42. Raw images of whole cells (**top panel**) and of magnified dendrites (**middle panel**) and reconstructed images of dendrites (**bottom panel**) of cultured hippocampal neurons under various experimental conditions are shown. *Stil* shRNA #1 with wild-type Rac1 (*Stil* kd + Rac1 WT), *Stil* shRNA #1 with constitutively active Rac1 (*Stil* kd + Rac1 V12), *Stil* shRNA #1 with wild-type Cdc42 (*Stil* kd + Cdc42 WT), or *Stil* shRNA #1 with constitutively active Cdc42 (*Stil* kd + Cdc42 V12) was introduced into neurons. The spines and dendritic shafts are visualized in blue and pink, respectively, in the reconstructed images. Scale bars = 10 µm (**top panel**) and 2 µm (**bottom panel**). (**B**) Distribution of dendritic spine density (number of spines per 10 µm of dendrite) under each condition. The data for the control and *Stil* kd alone are the same as those shown in Figure 3C. Each dataset was obtained from dendrites in neurons analyzed in at least three experiments. Mean values: 10.721 (control, *n* = 17), 2.877 (*Stil* kd, *n* = 19), 10.048 (*Stil* kd + Rac1 WT, *n* = 20), 10.357 (*Stil* kd + Rac1 V12, *n* = 26), 8.853 (*Stil* kd + Cdc42 WT, *n* = 25), and 10.454 (*Stil* kd + Cdc42 V12, *n* = 26). *** *p* < 0.001 Data were obtained from three independent experiments.

It was suggested that local activation of Rac by a guanine nucleotide exchange factor, ARHGEF7, at the synapse is required for spine formation [16]. Based on the findings that STIL associated with ARHGEF7 in the spine and was required for spine formation/maintenance in cultured neurons, we speculated that STIL is required for the activation of the ARHGEF7/Rac/PAK signaling pathway. Therefore, we wondered if the activation of Rac1 and/or Cdc42, which are known targets of ARHGEF7 and are involved in spine formation/maintenance, could compensate for the lack of STIL in spine formation. To this end, we performed rescue experiments on *Stil* knockdown neurons by introducing constitutively active Rac1 or Cdc42, as well as their wild-type counterparts; the introduction of constitutively active Rac1 or Cdc42 restored spine formation in *Stil* knockdown neurons to levels comparable to neurons without the knockdown, as expected (Figure 4A,B, *Stil* kd + Rac1 V12, *Stil* kd + Cdc42 V12). These results indicate that active Rac1/Cdc42 compensates for the reduction in spines caused by *Stil* knockdown. The fact that the overexpression of wild-type Rac1/Cdc42 also restored spine formation was enigmatic and unexpected (Figure 4A,B, *Stil* kd + Rac1 WT, *Stil* kd + Cdc42 WT). If only the constitutively active Rac, and not the wild type, had rescued the *Stil* knockdown-induced reduction in the dendritic spine, it would have strongly supported the hypothesis that phenotype is mediated by impaired Rac activation. We speculate that even if activation by ARHGEF7/STIL is insufficient, the presence of excess amounts of Rac1/Cdc42 may increase the number of their active forms such that it is sufficient for spine formation. In this regard, the fact that wild-type Cdc42 had a slightly lower rescue effect than constitutively active Cdc42 is noteworthy, although the difference was not statistically significant. In any case, the above rescue experiments provided evidence that overexpressed Rac1/Cdc42 can compensate for the reduction in spines due to *Stil* knockdown, but they did not provide clear evidence that STIL is necessary for Rac activation in the context of spine formation.

### 3.4. STIL Is Required for Rac Activation in Dendrites

**Figure 5:**
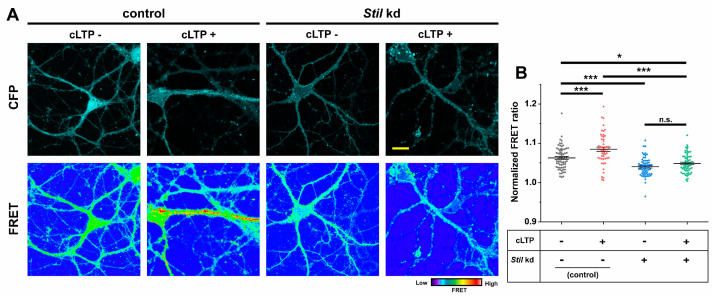
*Stil* knockdown impairs Rac activation in cultured hippocampal neurons. Raichu-Rac1, a bioindicator of Rac activity, was introduced into control and *Stil* kd neurons, FRET ratios were visualized, and FRET efficiencies were calculated. (**A**) Representative images showing CFP signal (upper panels) and FRET ratio (YFP/CFP; lower panels) in cultured hippocampal neurons harboring Raichu-Rac1. Control neurons with and without cLTP induction (cLTP+ and cLTP−, respectively), along with *Stil* kd neurons with and without cLTP induction, are shown. Scale bar = 5 µm. (**B**) Distribution of normalized FRET ratio under each condition. Each dataset was obtained from dendrites in neurons analyzed in at least three experiments. Mean values: 1.063 (control, cLTP−, *n* = 67), 1.085 (control, cLTP+, *n* = 53), 1.040 (*Stil* kd, cLTP−, *n* = 63), and 1.049 (*Sti*l kd, cLTP+, *n* = 59). * *p* < 0.05, *** *p* < 0.001, and n.s.: not significant.

Since the rescue experiments did not provide evidence that STIL is required for Rac activation in neurons, we introduced Raichu-Rac1, which visualizes Rac activity by means of fluorescent resonance energy transfer (FRET), into neurons to determine whether *Stil* knockdown affects Rac activation. As shown in Figure 5A,B, the basal Rac activity indicated by the FRET ratio/efficiency of *Stil* knockdown neurons (*Stil* kd, cLTP−) was lower than that of control neurons (control, cLTP−), which suggested that the dendritic spine reduction in *Stil* knockdown neurons was the consequence of impaired Rac activation.

Activation of the ARHGEF7/Rac/PAK signaling pathway has also been implicated in cLTP-induced spine formation and expansion in cultured hippocampal neurons [17,18]. Therefore, we examined whether *Stil* knockdown also affects Rac activation triggered by cLTP induction. As shown in Figure 5A,B, the FRET ratio/efficiency was significantly higher in control neurons with cLTP induction (control, cLTP+) than that in control neurons without induction (control, cLTP−). In contrast, the FRET ratio/efficiency of *Stil* knockdown neurons with cLTP induction (*Stil* kd, cLTP+) was not significantly different from that of *Stil* kd neurons without the induction (*Stil* kd, cLTP−). These results revealed that STIL is also required for cLTP-induced Rac activation, suggesting that STIL is also involved in activity-dependent synaptic plasticity.

### 3.5. cLTP Induces STIL Accumulation in Dendritic Spines and Promotes Its Association with ARHGEF7

**Figure 6:**
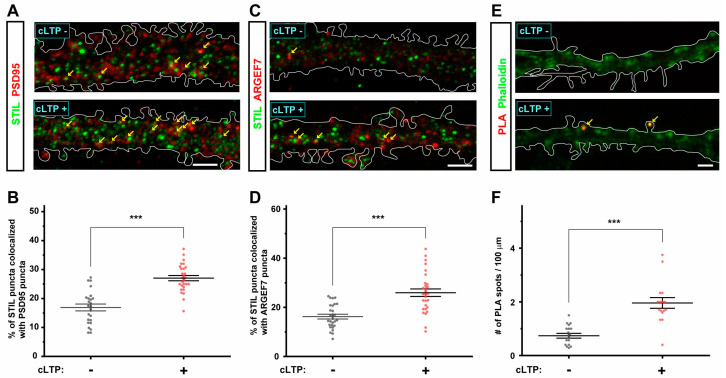
cLTP induces the accumulation of STIL in spines and promotes the association of STIL with ARHGEF7. (**A**) Representative images showing the distribution of STIL (green) and PSD95 (red) in the dendrite of cultured hippocampal neurons with and without cLTP induction (cLTP+ and cLTP−, respectively). The dendrites are outlined with white lines. Co-localization of STIL and PSD95 signals is indicated by arrows. Scale bar = 5 µm. (**B**) Ratios of STIL puncta co-localizing with PSD95 puncta among all STIL puncta in the dendrite of cultured hippocampal neurons with and without cLTP induction. Mean values: 16.902 (cLTP−, *n* = 24) and 27.042 (cLTP+, *n* = 28). (**C**) Representative images showing the distribution of STIL (green) and ARHGEF7 (red) in the dendrite of cultured hippocampal neurons with and without cLTP induction. The dendrites are outlined with white lines. Co-localization of STIL and ARHGEF7 signals is indicated by arrows. Scale bar = 5 µm. (**D**) Ratios of STIL puncta co-localizing with ARHGEF7 puncta in the dendrite of cultured hippocampal neurons with and without cLTP induction. Mean values: 16.219 (cLTP−, *n* = 27) and 25.943 (cLTP+, *n* = 29). (**E**) Representative images showing PLA signal spots (red) in the dendrite of cultured hippocampal neurons with and without cLTP induction. Spines are stained with phalloidin (green). The dendrites are outlined with white lines. PLA signals in the spine are indicated by arrows. Scale bar = 2 µm. (**F**) PLA signal spot density (numbers of PLA signal spots/100 µm) in the dendrite of cultured hippocampal neurons with and without cLTP induction. Mean values: 0.737 (cLTP−, *n* = 19) and 1.959 (cLTP+, *n* = 16). Each dataset shown in (**B**,**D**,**F**) was obtained from dendrites in neurons analyzed in at least three experiments. *** *p* < 0.001.

The results presented above indicate that STIL associates with ARHGEF7 in the dendritic spine and is required for spine formation/maintenance. The results also suggest that the requirement of STIL in spine formation/maintenance is dependent on its association with ARHGEF7. Therefore, we reasoned that the accumulation of STIL in the spine and its association with ARHGEF7 would be promoted when cLTP triggers spine formation and expansion. As shown in Figure 6A,C, STIL puncta themselves, especially the larger ones, appeared to be more abundant in dendrites after cLTP induction (cLTP+) than in dendrites without cLTP induction (cLTP−). We calculated the ratios of STIL puncta co-localizing with PSD95 puncta among all STIL puncta and found that the mean ratio in dendrites with cLTP induction (cLTP+) was higher than that in dendrites without cLTP induction (cLTP−) (Figure 6B), which indicated that STIL accumulated in the dendritic spine after cLTP induction. We then determined whether cLTP induction promotes the association of STIL with ARHGEF7; first, we examined the effect of cLTP induction on the distribution of ARHGEF7 as well as STIL and found that ARHGEF7 puncta, like STIL puncta, appeared more abundant in dendrites with cLTP induction (cLTP+) compared to those in dendrites without cLTP induction (cLTP−) (Figure 6C). The mean ratio of STIL puncta co-localizing with ARHGEF7 puncta among all STIL puncta in dendrites with cLTP induction (cLTP+) was higher than that in dendrites without cLTP induction (cLTP−) (Figure 6D). Finally, the PLA experiment revealed that the association of STIL with ARHGEF7 in the spine was promoted after cLTP induction (Figure 6E,F); PLA signal spots in spines were more evident in dendrites with cLTP induction (cLTP+) than in dendrites without cLTP (cLTP−), as shown in Figure 6E. The mean PLA spot density of neurons with cLTP induction (cLTP+) was more than twice that of neurons without cLTP induction (cLTP−) (Figure 6F). Thus, the results above indicate that STIL accumulation in the spine and its association with ARHGEF7 were enhanced by cLTP induction. The dynamics of these proteins after cLTP induction were consistent with the idea that the association between STIL and ARHGEF7 is required for spine formation/maintenance.

### 3.6. Stil Knock Down Results in the Reduction in Dendritic Spine Density In Vivo

**Figure 7:**
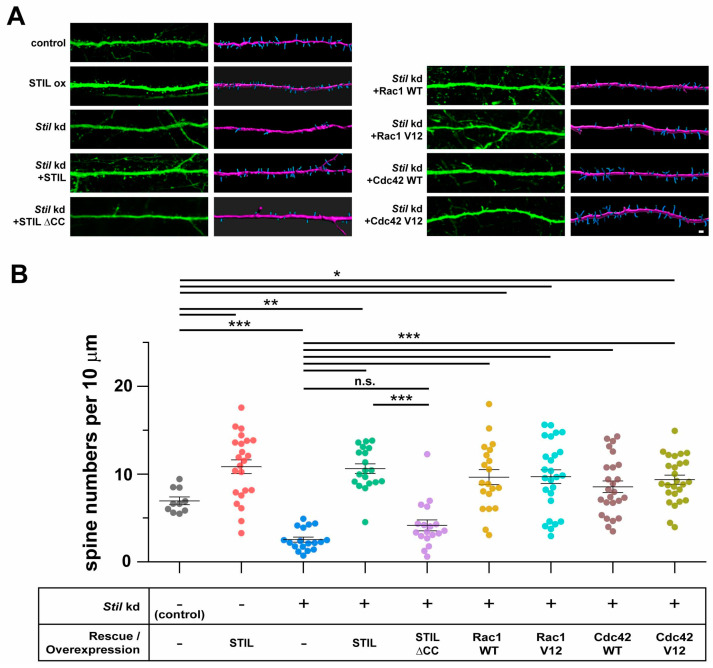
In vivo *Stil* knockdown causes dendritic spine reduction in cerebral cortical neurons, which is rescued by knockdown-resistant human STIL but not by human STIL lacking the coiled coil domain. Rac1 and Cdc42 induction can compensate for the reduction in dendritic spines caused by in vivo *Stil* knockdown. (**A**) Representative images of dendrites of a neuron overexpressing STIL and *Stil* knockdown neurons introduced with/without various rescue vectors. Each image set shows a raw image (**left**) and a reconstructed image (**right**) of a dendrite of a mouse cortical neuron at P30. EGFP alone (control), STIL (STIL ox), *Stil* shRNA (*Stil* kd), *Stil* shRNA with knockdown-resistant STIL (*Stil* kd + STIL WT), *Stil* shRNA with STIL DCC (*Stil* kd + STIL ∆CC), *Stil* shRNA #1 with wild-type Rac1 (*Stil* kd + Rac1 WT), *Stil* shRNA #1 with constitutively active Rac1 (*Stil* kd + Rac1 V12), *Stil* shRNA #1 with wild-type Cdc42 (*Stil* kd + Cdc42 WT), or *Stil* shRNA #1 with constitutively active Cdc42 (*Stil* kd + Cdc42 V12) was introduced into E14.5 neural progenitors by IUE. The spines and dendritic shafts are visualized in blue and pink, respectively, in the reconstructed images. Scale bar = 5 µm. (**B**) Distribution of dendritic spine density (number of spines per 10 µm of dendrite) under each condition. The dendrite morphologies displayed are representative of those observed in several sections of at least four brains from each electroporation group. Two sections including GFP-positive cells were analyzed. Mean values: 6.949 (control, *n* = 10), 10.837 (STIL ox, *n* = 22), 2.534 (*Stil* kd, *n* = 18), 10.619 (*Stil* kd + STIL, *n* = 19), 4.151 (*Stil* kd + STIL ∆CC, *n* = 18), 9.646 (*Stil* kd + Rac1 WT, *n* = 20), 9.711 (*Stil* kd + Rac1 V12, *n* = 26), 8.553 (*Stil* kd + Cdc42 WT, *n* = 25), and 9.359 (*Stil* kd + Cdc42 V12, *n* = 26). * *p* < 0.05, ** *p* < 0.01, and *** *p* < 0.001. n.s., not significant.

All the above data obtained from cultured neurons strongly suggested that STIL would be essential for spine formation/maintenance in vivo. To confirm this, we performed in vivo STIL overexpression and *Stil* knockdown experiments using IUE. Various constructs, similar to those used in in vitro studies, were introduced into neural progenitors in the brains of E14.5 mice and were used to analyze the spine density of their progeny at P30. As shown in Figure 7A,B, the results obtained from in vitro experiments were reproduced in vivo; *Stil* knockdown (*Stil* kd) resulted in a significant reduction in dendritic spines in mature neurons in the brain, which was restored by the introduction of shRNA-resistant STIL or various types of Rac. The mean dendritic spine density of *Stil* knockdown neurons in vivo was about 36% of that of control neurons. Intriguingly, regardless of whether endogenous *Stil* was knocked down or not, neurons expressing exogenous STIL (SITL ox, *Stil* kd + STIL) retained more spines than control neurons, which was different from the in vitro results. Human STIL without the CC domain (*Stil* kd + STIL ∆CC) lost their rescue effect to a great extent, similar to the results in cultured neurons. Neurons expressing various types of Rac retained more spines than control neurons, as did neurons expressing exogenous STIL (*Stil* kd + Rac WT, Rac1 V12, Cdc42 WT, and Cdc42 V12). However, only the difference between neurons expressing wild-type Cdc42 and control neurons was not statistically significant. These results confirmed that STIL is essential for spine formation/maintenance in vivo. Furthermore, the results indicated that excess amounts of STIL, as well as wild-type and constitutively active forms of Rac, enhance spine formation/maintenance in vivo, unlike the in vitro scenario. Thus, the amounts of STIL, as well as Rac, seem to be positively correlated with the degrees of in vivo spine formation. Physiological conditions possibly allow STIL to exert its positive effects on spine formation, which was not observed in in vitro experiments lacking sufficient intrinsic and extrinsic environments.

## 4. Discussion

In this study, we revealed that STIL is located in the dendritic spine and is associated with ARHGEF7 in cultured neurons and that *Stil* knockdown resulted in significant dendritic spine reduction both in vitro and in vivo. Our data further indicated that STIL is required for spine formation/maintenance in a CC domain-dependent manner and that STIL is required for Rac activation in dendrites. Furthermore, STIL accumulation in the spine and its association with ARHGEF7 appeared to be promoted by cLTP induction, which triggers the activation of the ARHGEF7/Rac/PAK pathway in the spine [17]. Thus, STIL and its dynamics were associated with the activation of the ARHGEF7/Rac/PAK signaling pathway, suggesting that STIL was required for spine formation/maintenance via this pathway. As a result, STIL has emerged as a novel regulator of spine morphogenesis.

STIL is one of the centrosomal proteins and, together with its binding partners PLK4 and SAS-6, constitutes the core module for centriole duplication [19,20,21]. In particular, the association and complicated mutual regulation of PLK4 and STIL at the circumference of the mother centriole are considered key initial steps in ensuring the biosynthesis of a single daughter centriole [19,20,22]. Interestingly, genes encoding STIL and SAS-6 have been designated as *MCPH7* and *MCPH14*, respectively; MCPH stands for “MicroCephaly Primary Hereditary” characterized by a small brain size at birth due to putative genetic causes. Currently, at least 27 genes are listed as the *MCPH* genes in the Online Mendelian Inheritance in Man (OMIM) database [23], of which at least 8, including *STIL* and *SAS-6*, have established roles in centriole duplication [23,24,25]. In addition, variants in the *PLK4* gene have been identified in patients with autosomal recessive microcephaly accompanying chorioretinopathy [26,27]. These facts imply that dysregulated centriole duplication results in impeded cortical neurogenesis during development, and the knockout of the *Sas-4* gene (also known as *Cenpj*, or CRAP and *MCPH6* in humans) in neural stem cells was reported to cause microcephaly in mice [28]. However, direct evidence for the involvement of STIL in cortical neurogenesis remains to be provided since global *Stil* KO mice were embryonic lethal at E10.5 before active cortical neurogenesis took place [29].

In addition to their crucial role in cell division and proliferation, centrioles that serve as basal bodies are also essential for ciliogenesis in non-dividing cells [30]. An absence of primary cilia mediating Sonic Hedgehog (SHH) signaling was reported in *Stil* KO mice and was considered a cause of embryonic lethality [29,31]. In addition, STIL associates with SUFU and GLI1, which are downstream mediators of SHH signaling, suggesting that STIL is directly involved in the regulation of SHH signaling [10,32]. More recently, we found that STIL is involved in the regulation of cell motility via direct association with ARHGEF7 in cancer cells [14].

Considering that STIL has such a fundamental role in a variety of cellular activities, it is not surprising that it shares an important role in post-mitotic neurons. Although a pioneering study on the *STIL* gene reported that *STIL/Stil* mRNA was undetectable in human and murine brain [33], contemporary RNA seq data provided by the Genotype-Tissue Expression (GTEx) project “https://www.gtexportal.org/home/aboutGTEx (accessed on October 2024) indicated very low but ubiquitous expression of *STIL* mRNA in human tissues, and the present study confirmed widespread cytoplasmic STIL distribution in cultured mouse neurons. Interestingly, Carr et al. also reported that STIL located in outgrowing neurites of differentiated mammalian dopaminergic PC12 cells and predicted further novel functions for STIL in post-mitotic cells [34]; the present finding that STIL is involved in and essential for the regulation of dendritic spine formation in neurons is an example of such functions. However, it does not exclude the possibility that STIL may have additional roles in post-mitotic neurons since STIL signals in dendrites were not confined to the spine, not to mention their distribution in the soma (Figure 1).

The detailed molecular mechanism underlying the regulation of spine formation/maintenance by STIL is currently unknown. However, the mechanistic function of STIL in centriole duplication may provide clues, although it is not fully understood and is still under investigation. Recent studies on Ana2, the fly homolog of vertebrate STIL, have revealed that Ana2 proteins form tetramers and that Ana2, together with Sas-6 and Sas-4 (mammalian CRAP/CENPJ), are self-organized into macromolecular structures partially mimicking centriole assembly [35,36]. These findings suggested that STIL is a structural protein serving as a scaffolding protein, and indeed, STIL is known to have multiple domains required for interaction with other centrosomal proteins [37]. The CC domain, which we found to be crucial for spine formation/maintenance, is one such domain and mediates not only the association with ARHGEF7 [14] but also the oligomerization of STIL [38] and the association with PLK4 [21]. Therefore, the requirement of the CC domain for spine formation/maintenance cannot be attributed solely to mediating the association with ARHGEF7. Rather, the CC domain can be important as a key domain for STIL-mediated macromolecular construction. Intriguingly, relatively large STIL puncta were found to increase after cLTP induction, which may be a manifestation of macromolecular construction. Precise observation of STIL puncta in the spine with a super-resolution microscope would provide further information.

As for the target Rho GTPases of ARHGEF7, Rac1 and Cdc42 were identified many years ago [39]. Since these two members of the Rho GTPase family have long been known to induce plasma membrane protrusions named lamellipodia and filopodia by regulating actin dynamics [3], their role in spine formation/maintenance is also of great interest. Several studies have been conducted to introduce constitutively active and/or dominant-negative Rac into cultured neurons. These studies revealed that, in general, the excessive activation of Cdc42 has no effect or only a slight effect on spine formation/maintenance [40,41,42], while the excessive activation of Rac1 induces numerous incomplete spines or dendritic protrusions [8,40,42,43]. Conversely, the inactivation of Cdc42 or Rac1 by the introduction of dominant-negative forms generally resulted in a decrease in spines in primary cultured neurons [8,41,42,43,44]. The finding that spines are decreased in primary rat hippocampal neurons with the knockdown of Cdc42 or Rac1 further indicated that Cdc42 and Rac1 are independently required for spine formation [44,45]. Thus, Cdc42 and Rac1 themselves, and their proper activation, are considered essential for spine dynamics by regulating actin polymerization, and they appear to play a unique role in this process. However, the precise respective roles that Cdc42/Rac1 play in various aspects of spine dynamics, including the initial formation, maturation, and stabilization of spines, are not yet fully understood. In addition, it has also not been clarified which, or both, are actually activated by ARHGEF7 during the formation/maintenance of the spine. We expected that rescue experiments with constitutively active and wild-type Cdc42/Rac1 would provide clues as to which GTPase is primarily affected by *Stil* knockdown. However, not only constitutively active forms but also wild-type Cdc42 and Rac1 similarly compensated for the *Stil* knockdown-induced reduction in spines. This seems to be incompatible with our speculation that the reduction in spines was mediated by impaired Rac activation. However, as noted above, previous studies have shown that the introduction of constitutively active Cdc42 has no effect or a small effect on the number of spines formed in cultured neurons [40,41,46]. Thus, spine formation induced by constitutively active Cdc42 appears to be specific to the *Stil* knockdown situation, suggesting that Cdc42 is a downstream signaling molecule in spine formation/maintenance mediated by STIL/ARHGEF7. This may be supported by the fact that wild-type Cdc42 appeared to be less effective in restoring spines than constitutively active Cdc42, both in vitro and in vivo, although the difference was not statistically significant. Further studies are necessary to elucidate the precise regulatory pathways involved.

This study was initiated by the finding that STIL is associated with ARHGEF7 in the spine. However, PLA signals representing the association were very rare and were found in only a few spines per single neuron. Even under conditions where spine formation/expansion was evoked by cLTP induction, the number of the signals was not abundant; yet, it was clearly higher than that in the basal state. These results indicate that the association between STIL and ARHGEF7 is transient rather than persistent. In this context, it is surprising that *Stil* knockdown resulted in significant spine reduction, which suggests that spine formation/maintenance is highly dependent on such a transient association. We deduce that the association of STIL with ARHGEF7 is only required when the activation of the ARHGEF7/Rac/PAK signaling pathway is initiated. However, it is possible that the local insufficiency of STIL is not the only cause of spine reduction. As mentioned above, STIL is necessary for ciliogenesis [31,47], and Kumamoto et al. revealed that primary cilia of hippocampal dentate granule cells are necessary for them to receive synaptic input [47]. Although they did not examine whether the spine density was reduced, the impaired synaptogenesis may have been accompanied by a reduction in spines. Since the significance of diverse and generally crucial cilia-mediated signaling in mature neurons is not fully understood, we cannot exclude the possibility that the reduction in spines due to global *Stil* knockdown is also mediated by impaired ciliogenesis. Additionally, ARHGEF6, another Rac-GEF that is highly similar to ARHGEF7 and has also been implicated in spine morphogenesis, may be involved in STIL-mediated spine formation [48,49]. Further studies are needed to fully understand how this centrosomal protein contributes to spine dynamics.

## Figures and Tables

**Table 1:** PCR primers and shRNAs used in the present study.

Application	Nucleotide Sequence (5′—3′)
Forward qPCR primer for *Actb*	ACCTTCTACAATGAGCTGBCG
Reverse qPCR primer for *Actb*	CTGGATGGCTACGTACATGG
Forward qPCR primer for *Stil*	AGGTATGGGCTTGCTGCTTGAGAT
Reverse qPCR primer for *Stil*	GTAACTGAAACTGAAGGTCGGGCG
Forward PCR primer for *STIL* cloning	ATGTACCCATACGATGTTCCAGATTACGCTAATACCAGGTTTCCTTCAAGCAAGATGG
Reverse PCR primer for *STIL* cloning	GAACCCTTAAACATGTTAAAATAACTTAGGTAACTGTCTGAGACGCTTTACG
*Stil* knockdown shRNA #1	GCCGCAGCTAATCAAGTTCA
*Stil* knockdown shRNA #2	CAAGTTCAGGGAACCTATAA

## Data Availability

Data are available from the corresponding author upon reasonable request and with the permission of Dr. Tohru Matsuki and Dr. Atsuo Nakayama.

## Supplementary Materials

The following supporting information can be downloaded at: https://www.mdpi.com/article/10.3390/cells14020062/s1, Figure S1: Endogenous STIL and ARHGEF7 represent weak interaction within the synaptosome. Immunoblot analysis of STIL detected only a modest interaction with ARHGEF7 (lane 2, asterisk).
