# Supplementary material for: The MCPH7 Gene Product STIL Is Essential for Dendritic Spine Formation
Source: Cells. 2025 Jan 7;14(2):62. doi: 10.3390/cells14020062 (PMC11764357; doi:10.3390/cells14020062)
Supplement: Supplementary file 1 [file cells-14-00062-s001.zip › cells-3359478-Figure S1.pdf]

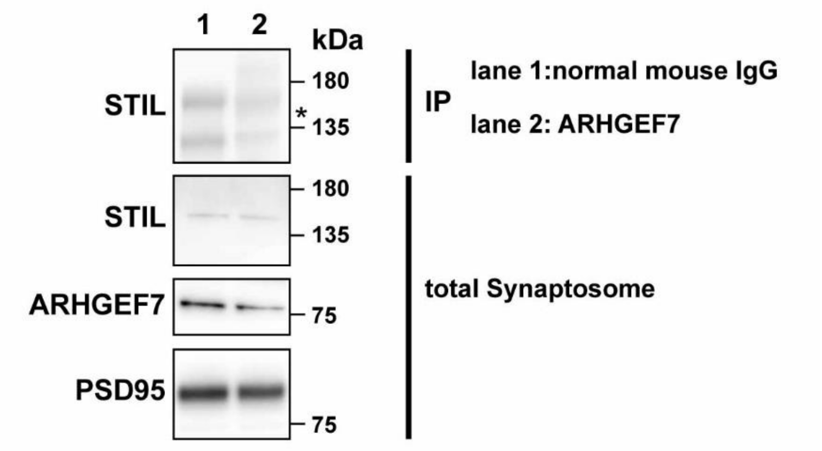

Figure S1 Endogenous STIL and ARHGEF7 represent weak interaction within the synaptosome. Immunoblot analysis of STIL detected only a modest interaction with ARHGEF7 (lane 2, asterisk).

Materials and Methods

Synaptosome was purified from 300 mg of cortex isolated from wild type ICR mice at postnatal day 60 (Japan SLC, Hamamatsu, Japan) using Minute™ Synaptosome Isolation Kit (Cat#SY-052, Invent Biotechnologies, Plymouth, MN, USA). Isolated synaptosome was dissolved with 1% NP40 including solution (NP40-IPB; 137 mM NaCl, 2.7 mM KCl, 1% IGEPAL CA-630, 25 mM Tris-HCl [pH7.4], 2 mM EDTA, 50 mM NaF, 0.1% 2-mercaptoethanol, protease inhibitor cocktail [cOmplete Mini, EDTA-free; Roche], 1 mM sodium orthovanadate, and 1 mM phenylarsine oxide) and divided into two tubes for further immunoprecipitation. Immunoprecipitation was performed using anti-ARHGEF7 antibody (Cat# sc-393184, Santa Cruz Biotechnologies, Dallas, TX, USA) and normal mouse IgG. Each immunoprecipitation was performed using SureBeads Protein G Magnetic Beads (Cat# 1614023, Bio-Rad, Hercules, CA, USA). Immunoblot of STIL, ARHGEF7, and PSD95 were performed using anti-STIL [1], anti-ARHGEF7 (Santa Cruz), and anti-PSD95 antibodies (Cat# K28/43, UC Davis/NIH NeuroMab Facility, Davis, CA 95616, USA).

Reference

1. Kasai, K.; Inaguma, S.; Yoneyama, A.; Yoshikawa, K.; Ikeda, H., SCL/TAL1 interrupting locus derepresses GLI1 from the negative control of suppressor-of-fused in pancreatic cancer cell. *Cancer research* **2008,** 68, (19), 7723-9.
